# Indigenous sovereignty in digital territory: a qualitative study on land-based relations with #NativeTwitter

**DOI:** 10.1177/11771801211019097

**Published:** 2021-07-02

**Authors:** Ashley Caranto Morford, Jeffrey Ansloos

**Affiliations:** 1 Department of Liberal Arts, Pennsylvania Academy of Fine Arts, Philadelphia, Pennsylvania, United States; 2 Department of Applied Psychology and Human Development, University of Toronto, Canada

**Keywords:** land-based relations, Indigenous language revitalization, social media, Twitter, Indigenous technology, Indigenous new media

## Abstract

Technology scholars have often framed cyberspace as landless. Critical technology and Indigenous new media scholars have critiqued this approach, citing the land-based nature of Internet infrastructure. This study seeks to further develop the conceptual framework of Indigenous land-based relations through qualitative analysis of Indigenous language revitalization networks within Twitter. Using a thematic analysis approach, six key themes emerged: (a) Land-based cyber-pedagogy, (b) Rematriations of land-based relations in digital environments, (c) Digital bridges to homelands and lifeways, (d) Networked cultural navigation, (e) Settler colonialism in cyberspace, and (f) Indigenous digital sovereignty and cyber-justice. Implications for theory and practice in both new media studies and language revitalization are considered, with a focus on elucidating the land-based nature of the Internet, Indigenous people’s navigation of colonialism within the Internet, and the meaning of anti-colonial resistance in cyberspace.

## Introduction

In the history of the Internet, conceptualizations of the Internet have largely been framed as landless. Such imaginings portray the Internet and Internet technologies as devoid of land-based relations. When we speak of land-based relations, we are referring to the rooted contexts of life; the material, spiritual, and imagined links between peoples—human and non-human—and places; the connections between land-based materials and peoples’ uses of these materials; and the comprehensive politics and ethics of place and space as they intersect with land. In speaking of these relations, we recognize the endless, ongoing ways that humankind learns “both *from* [. . .] and *with* the land” (Simpson, 2014, p. 7), we understand that land and the self are in deep relationship (Styres, 2017, p. 88), and we know that “land is sacred, spiritual, experiential, and expressive” (p. 88).

While theorizations of the Internet have evolved to encompass clearer understandings of the potential for human connections vis-à-vis cyberspace, the environment of cyberspace itself has largely continued to be framed as landless (Duarte, 2017; Gaertner, 2016). This framing is likely because, as it relates to the politics of place, the Internet is predominantly seen as universal and democratic (Nakamura, 2002). This history devoid of land-based thinking is inaccurate, and it substantially limits how we might understand Indigenous peoples’ lives as they intersect with cyberspace. Simpson (2017) emphasizes, “land-based relationships are the foundation of Indigenous thought” (p. 213). Recent scholarship at the intersections of Indigenous, critical race, and new media studies has posited the vitalness of developing theory and documentation of the inextricable relationships between land and digital technologies (Duarte, 2017; Gaertner, 2016; Loft & Swanson, 2014; Wemigwans, 2018).

Conceptual development is needed to understand (a) the land-based nature of the Internet, (b) Indigenous peoples’ navigation of colonial dynamics fraught in the structure and experience of cyberspace, and (c) the meaning of decolonial resistance in cyberspace. We take up these challenges in the context of #NativeTwitter. #NativeTwitter refers to, and is a hashtag often used by, the many Indigenous people gathering on Twitter to collectively discuss Indigenous experiences, cultures, communities, research, stories, and life (Monkman, 2018). Through a qualitative analysis of an archive of interviews and tweets focused on Indigenous language revitalization, we consider how #NativeTwitter is informing theorizations of land-based relations in cyberspace, and how Indigenous tweeters are navigating and resisting the settler colonial dynamics of the Internet.

## Literature review

### Cyberspace and land

In this history of thought regarding cyberspace and land, representations of land have often been reductive, merely cast as setting to human enterprise, and rarely considered in terms of the dynamic sociopolitical, cultural, and relational context to human and more-than-human life. In the science fiction trilogy Sprawl (1984–1988), William Gibson began to imagine the possibilities of computer technologies now familiar within mainstream society (Lewis, 2014, p. 55). When the first novel of the series, *Neuromancer*, was published in 1984, it popularized the now well-known term “cyberspace” and began to transform how mainstream society perceived the ecology, potentials, and purpose of the Internet (Gaertner, 2015, para. 14; Lewis, 2014, p. 55). David Gaertner (2015) describes the cyberspace that *Neuromancer* presented as “numbers and bureaucracy [. . .] brightly coloured, quickly moving constellations of data that carry uncanny trace[s] of a city or a world [. . .] There are no residents in Gibson’s cyberspace, just tourists and hackers” (para. 16). Gibson’s cyberspace, with its bureaucracy and hacking threats, recognizes the Internet as a setting that can breed capitalism and violent infiltrations of trolls and computer viruses. With its uncanny resemblance of cityscapes and earthscapes, however, Gibson’s imagining hints at but fails to fully conceptualize the potentials of the Internet as a social, cultural, and political environment where life can connect, develop, and thrive.

As Gaertner (2015) posits, while Gibson’s novel was critical in shaping mainstream theories of the Internet, it was Neal Stephenson’s (1992) *Snow Crash* that transformed the vision of cyberspace—what *Snow Crash* termed “the metaverse”—more fully into a sociopolitical environment (Gaertner, 2015, para. 17). Noting the shift between *Neuromancer* and *Snow Crash*, Gaertner (2015) writes,

Stephenson’s cyberspace [. . .] is a setting for characters to wage wars, fall in love, or even build a home [. . .] a universe, with its own geopolitics and vested human interests. Unlike Gibson’s bureaucratic constellations of light, the metaverse is a social terrain. (paras. 17–18)

The social terrain of the Internet is perhaps most explicitly perceived through chat rooms, online forums, and social media platforms like Facebook, Instagram, and Twitter. Yet, while Stephenson’s conceptualization perceives cyberspace as reflective of and central to human life, his imagining is one that is landless. “The sky and the ground” of this cyberworld, *Snow Crash* tells us, resemble “a computer screen that hasn’t had anything drawn on it yet” (p. 24). The common assumption of cyberspace as landless does not grasp the Internet’s dependence on land to exist and survive. An Indigenous studies approach to technology reminds us of this crucial connection.

### Cyberspace, land, and settler colonialism

Indigenous theorizations of cyberspace, and utilizations of digital technologies to connect in cyberspace, clarify the land-based nature of the Internet, and the politicization of that territory. Activities, conversations, and mobilizing that occur in cyberspace can have concrete on-the-ground impacts, as witnessed in Indigenous rights movements like #IdleNoMore, #StandWithStandingRock, and #WetsuwetenStrong (Duarte, 2017; Jackson et al., 2020; Wemigwans, 2018). Jason Edward Lewis, a Cherokee (Indigenous peoples of the Southeastern USA), Hawaiian (Indigenous people of the Hawaiian Islands), and Samoan (Indigenous people of the Samoan Islands) scholar, describes cyberspace through land- and water-based conceptualizations—as a sea and “vast archipelago of websites, social media services, shared virtual environments, corporate data stores, and multiplayer video games” (pp. 56, 58). Similarly, Joanna Hearne (2017) draws on “an array of land-based metaphors for digital media—the web, the rhizome, and the river” to describe and emphasize the imaginative place-based conceptualizations and possibilities of Indigenous digital studies (p. 8). And Pascua Yacqui (Indigenous people of Southwestern USA) scholar Marisa Elena Duarte’s (2017) *Network Sovereignty* emphasizes, “the place-based nature of the Internet” (p. 8) when she writes that

[i]t is perhaps easy for city-living folks to imagine the Internet as something “out there,” as invisible and ephemeral as droplets of water in the air we breathe, and with data centers and network operations as nondescript as the next strip mall. It is more realistic for tribal residents to conceptualize the Internet as something “right here,” with decisions about where to build towers shaped by seasonal rhythms of hunting, wildfires, and prayer, not to mention the matter of land and edifice allocation. (pp. 53–54)

Internet functionality requires land-based infrastructures. The relationship between the digital and land is inextricable, and it is erroneous to think of cyberspace as landless.

Duarte also recounts how corporate construction of digital infrastructures has displaced and dispossessed Indigenous peoples of their homelands and right to self-determination. This recounting highlights the interconnections between cyberspace and land, including the settler colonialism entwined within cyber-systems. In that vein, we return to Stephenson’s description of the metaverse in *Snow Crash.* That his cyberworld “hasn’t had anything drawn on it yet” echoes settler colonial claims that Indigenous lands were terra nullius for White people to take (Stephenson, 1992, p. 24). Nor can we overlook how Gibson and Stephenson’s novels portray the racial territory of cyberspace. *Neuromancer*’s world is centred on a White protagonist and, while Stephenson’s novel features Indigenous and racialized characters, including the Aleut (Indigenous peoples of Aleutian Islands in Alaska, USA) character Raven, they are stereotyped and uphold racist tropes. These novels craft cyberspace as a White territory.

With the rise of computer-based technology, settler colonialism has seeped into the cyber-realm. The Internet has become another space and place where the violence and normalization of colonization are perpetuated. Black, Indigenous, and scholars of colour—including Loretta Todd, who is Cree (Indigenous people of northern plains region of North America) and Métis (Indigenous people of the plains of Canada), Lisa Nakamura, Safiya Noble, and Marisa Elena Duarte—have recognized that the Internet widely operates as an extension of the colonial and Eurocentric world. L. Todd (1996) has pointed out that, as the Internet has been developed through Western epistemologies, it replicates the oppressiveness of colonial society through the protocols on which it is built, and the social interactions that are supported and enabled by such protocols. Nakamura (2002) recognizes the Internet as a space both shaped by and re-shaping normative White of race and ethnicity. Platforms like Twitter, Facebook, and YouTube mirror hegemonies of Whiteness enacted on marginalized bodies in analogue spaces. Noble (2018) has shown that racism and sexism are embedded within the algorithms of search engines, and the sociopolitical complicity that hubs of technology development have in maintaining status quos of colonial race and gendered relations. In drawing attention to colonial rhetoric commonly used to describe the Internet, Duarte (2017) writes that

[i]t is no coincidence that the discourse of Internet entrepreneurship is marked by the discourse of Manifest Destiny. Consider the terms and phrases *information wants to be free, Electronic Frontier Foundation, and Internet pioneer*. For Native peoples, it is as if the imperial urge to westward expansion moved into the cybersphere. (p. 113)

We also witness colonial logics playing out in social media: for instance, Twitter has an ongoing issue of suspending Indigenous, Black, and other racialized users when they speak out against racist tweets, while those who write the racist tweets often go unaddressed (Jackson et al., 2020). The extent to which these suspensions are algorithmically governed, or decisions made by human actors is unknown, but they mirror processes which occur in analogue environments.

Counter to these examples of virtual colonialism, Mohawk (Indigenous peoples of eastern North America) multi-media artist Skawennati and Jason Edward Lewis co-founded the Aboriginal Territories in Cyberspace (AbTec) research-creation network in 2005. AbTec’s purpose is to ensure that Indigenous life and sovereignty is present in the digital realm, including Internet spaces and virtual gaming. Lewis (2014) describes AbTec “as a vehicle for staking out Aboriginally determined territories within cyberspace” (pp. 59–60). AbTec’s research projects enable Indigenous people “to experiment with ways they and their communities might leverage digital media as a tool for preserving and advancing culture and languages, and for projecting a self-determined image out into a mediasphere awash in stereotypical portrayals of Native characters” (p. 64). Relatedly, Indigenous language revivalists like Cherokee technologist Joseph Erb et al. (2018) and Anishinaabe (Indigenous peoples of Great Lakes region of North America) scholars, Margaret Noodin and Stacie Sheldon (2017) are contending with how to respectfully extend Indigenous languages, including songs and writing systems, into the digital realm in culturally grounded, creative, and nourishing ways. And, building on these questions and explorations, Lewis, Kanaka Maoli (Indigenous Hawaiian) scholar Noelani Arista, Cree (Indigenous people of northern plains region of North America) scholar Archer Pechawis, and Oglala Lakota (Indigenous people of plains region of North America) scholar Suzanne Kite (Lewis et al., 2018) consider AI, algorithms, and other computer technologies as non-human kin, asking how Indigenous kinship epistemologies might guide us in living in respectful relationality with them.

#NativeTwitter functions as this type of community-based vehicle for engagement with complex questions about Indigenous life, human and non-human kin, and cultural resurgence. Dorothy Kim et al. (2018) recognizes that Twitter is a “space where [Black, Indigenous, and] communities of color can talk to each other and build their own worlds” (p. 151). Indigenous people are carving out a markedly Indigenous territory within Twitter. While Twitter is but one corner of the ever-evolving landscape of the Internet, and more specifically communications and social media platforms, it has played a crucial role in Indigenous cultures (Duarte, 2017; Wemigwans, 2018). While Indigenous cultural production is occurring elsewhere and differently in other mediums such as Facebook, TikTok, Instagram, and YouTube, our Twitter-based study elucidates important Indigenous conceptualizations of the Internet, particularly at the intersections of land. In understanding this intersection, we can better grapple with how Indigenous peoples are navigating both colonial experiences and anti-colonial movement.

## Methodology

Our study is situated within critical technology studies and Indigenous new media theories—namely, in the rejection of the landless framing of the Internet, and assertion that Indigenous peoples’ engagements with the Internet are land-based and imply the comprehensive politics of land-based relations. To develop this further, our study draws on an analysis of tweets obtained through the #DecolonizingDigital archive at the University of Toronto. This archive was created by Jeffrey Ansloos, with the help and insights of a team of undergraduate and graduate student researchers, including Ashley Caranto Morford. The archive creation was funded by the Social Sciences and Humanities Research Council in Canada and is accessible by request. The archive is comprised of digital materials and interviews produced from 2006 until present that highlight Indigenous contributions in social media environments, in the areas of Indigenous languages, artistic resurgence, and health information. While the materials collected focus on the Canadian context, given the transnational nature of the digital environment, and engagement of international users within Canadian origin hashtag networks, there is some content included that is produced beyond the geo-catchment of Canada. The archive from which our study was drawn consists of 8,820 tweets and 10 interview transcriptions with Indigenous Twitter users that were collected between the years of 2018 and 2019. Our study draws on a collection from this archive, *Language Learning and Resurgence* which focuses on Indigenous language revitalization in the Canadian context. Through this study, we engaged approximately 1,900 Twitter accounts, 9 account types, 35 hashtag networks, 57 keyword terms, and 3,812 tweet samples. The networks and keywords searches included in this archive are based on the federally recognized Indigenous languages within Canada, of which there over 60.

Acknowledging the interdisciplinary nature of our project and research backgrounds, we bridge methods of reading with Indigenous theory with qualitative thematic analysis of materials in order to elucidate key themes emerging within this archive. On the theoretical level, we were interested in understanding the land-based nature of the Internet as informed by Indigenous Twitter, Indigenous peoples’ navigation of colonialism embedded within Twitter, and the meaning of decolonization within this space. The analysis presented here is thematic and draws a contextualist approach (Braun & Clarke, 2006), attending to both the subjective lived experiences of individual Indigenous Twitter users as reflected in their tweets and interviews, as well as the broader social dynamics in which their digital activities occur.

Drawing on Braun and Clarke’s (2006) thematic analysis method, which has been used in both humanities and social science research, we engaged in a practice of repeated reading of the social media and interview materials. During this phase, we reviewed the archive of tweets and interviews to support familiarity with the materials and to take note of initial organizing structures and conceptual issues. We then used our initial notes to develop a list of codes and used NVivo to code each tweet and interview image-by-image, and interviews line-by-line. To account for continuity, coherence, and integrity in this coding process, we were committed to a consensus-based approach to reconciling differences and disagreements in our perspectives on individual tweets. Reconciliation is an important interpretative practice, inasmuch as it is often framed as a measure of rigour for scientific validity. In our study, the practice of reconciliation did not occur in order to make positivist claims to the validity of our interpretation, but rather, to highlight the strength of communal reading practices and the limits of individual interpretations. Following reconciliation, similar codes were organized into broader themes. We considered how these themes spoke to both individual lived experience and broader social dynamics. We developed descriptions of these themes and curated a selection of relevant examples from our archival analysis in order to provide a close reading and elucidate the analysis. Throughout the study, we also engaged in member-checking, which is a process whereby we engaged directly with Indigenous social media users who authored tweets to provide feedback on our interpretative process.

This approach to the thematic coding is foremost interpretative, deeply resonant with the practices of close reading and discourse analysis in Indigenous studies, and humanities more broadly. Our approach to thematic analysis, and the coding process inductive, adding to the contextual and interpretative aspects of tweets.

This study was approved by our university research ethics board, and data use and analysis were conducted in alignment with the Tri-Council Policy Statement on Ethical Conduct for Research Involving Humans particularly the standards for research involving First Nations, Inuit, and Métis Peoples in Canada. We also considered how to best approach the use of public domain Indigenous social media materials, particularly regarding the issue of ensuring the privacy of Twitter users. The standard set by most social media researchers is to anonymize social media content, but we recognize that it is easy to compromise user anonymity through several means. While all tweets in this archive are publicly available, we contend that their status as publicly visible does not equate to consent for research use. To address these issues, we adopted a standard that the tweets from the personal accounts of Indigenous users quoted and cited within this piece have either been previously published in other respected Indigenous studies spaces or are quoted with the direct permission of the Twitter user. We feel strongly that as researchers continue to engage within digital ecologies, and considerations of ethical issues emerge with regard to data use and stewardship, Indigenous studies scholars must be committed to upholding clear consent practices.

## Results

Across our analysis, we identified the following six key themes that contribute to a developing theoretical position on the land-based nature of the Internet, Indigenous peoples’ navigation of colonial dynamics of cyberspace, and the meaning of decolonial resistance and cyber-justice in cyberspace.

### Land-based cyber-pedagogy

**Figure 1.**
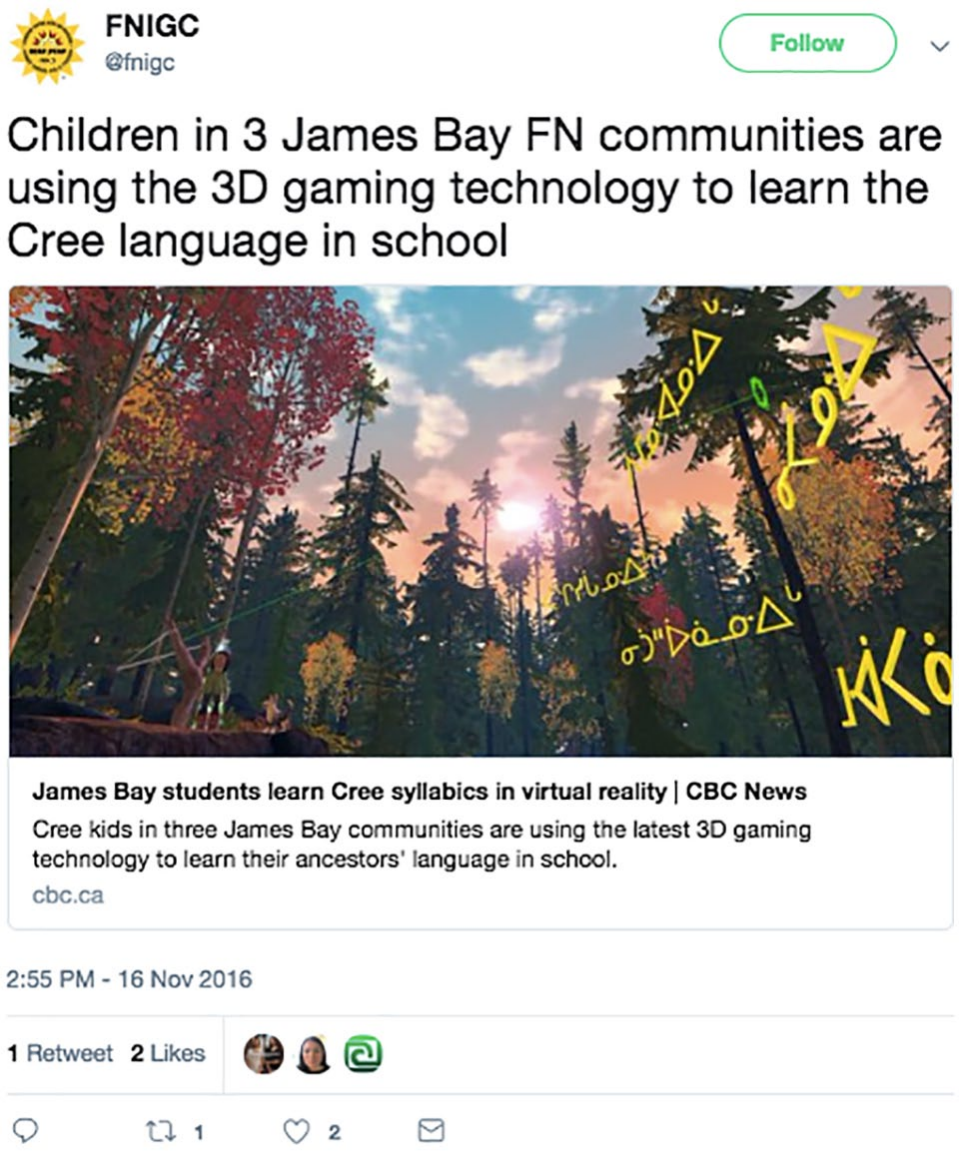
@fnigc tweets about how 3D gaming technology assists James Bay FN students learn Cree language in school. FN: First Nations; Cree: Indigenous people of northern plains region of North America.

Simpson (2014) emphasizes that Indigenous education must “come through the land” (p. 9). Indigenous language revivalists (Corntassel & Hardbarger, 2019; McIvor, 2009) talk about the importance of land-based learning to Indigenous language revitalization, given how intertwined languages are with the lands from which they have emerged. Mainstream discourse about the Internet as landless risks presenting or misconceiving online learning as inevitably disconnected from physical place. While the Internet cannot replace the experience of literally being on the land, language learning occurring within #NativeTwitter emphasizes that Indigenous peoples are practicing consciously land-based cyber-pedagogy—pedagogy that, though occurring online, is committed to teaching the connections between Indigenous lands and Indigenous languages. A tweet by @fnigc illustrates this type of land-based cyber-pedagogy, by showing how children in three James Bay First Nations communities are engaging with virtual reality technology that enables them to digitally move through vivid three-dimensional (3D) recreations of their homelands while they learn their language (Figure 1).

Indigenous land-based pedagogy is not absent from cyberspace. As these examples demonstrate, cyberspace provides a connective means by which Indigenous people are tethered in relationship to place, even in and through digital environs.

### Rematriations of land-based relations in digital environments

Simpson (2014) articulates that Indigenous conceptions of land include the spiritual world (p. 10). Not only do the language teachings on #NativeTwitter offer land-based learning, they do so in ways that are grounded in nation-specific spiritual and cultural knowledges connected to land and language. These tweets are rematriations not only of language but of land-based relations. As Newcomb (1995) suggests, processes of rematriation help “to restore a living culture to its rightful place on [and with] Mother Earth” and

to restore a people to a spiritual way of life, in sacred relationship with their ancestral lands [. . .] rematriation acknowledges that our ancestors lived in spiritual relationship with our lands for thousands of years, and that we have a sacred duty to maintain that relationship for the benefit of our future generations. (p. 3)

**Figure 2.**
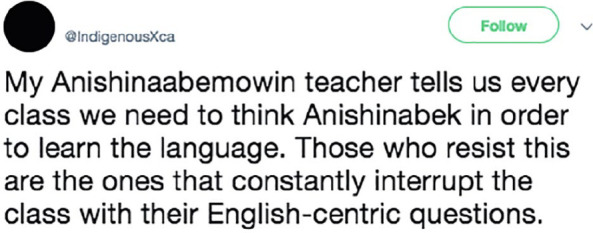
@IndigenousXca tweets about a language-learning tip for learning Anishinabemowin. Anishinaabemowin: the language of the Annishinaabe people; Anishinabek: the plural tense of the word Anninaabe, which refers to the Annishinaabe people, an Indigenous people in the Great Lakes region of North America.

Many language-based tweets understand and clarify the unbreakable connection between Indigenous lands, cultures, and peoples. By offering this understanding, Indigenous Twitter users enable language learners to not merely memorize words, but to learn how to embody and live their languages attentive to ancestral, ongoing relations and responsibilities to land. This sentiment is reflected in the tweeted insights of an @IndigenousXca account host who is learning Anishinaabemowin (the language of the Annishinaabe people, an Indigenous people in the Great Lakes region of North America) (Figure 2).

**Figure 3.**
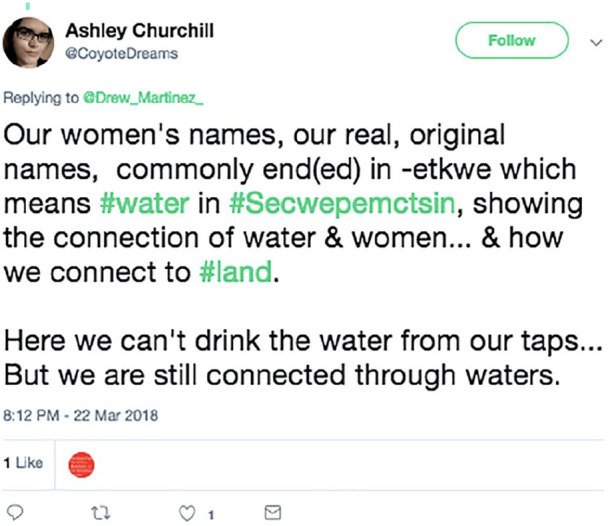
@CoyoteDreams tweets about women’s names and connectedness to water. Secwepemctsin: Indigenous people of interior region of British Columbia in Canada.

A tweet by @CoyoteDreams offers a concrete example of how Indigenous language-learning spaces on Twitter are filled with land-based and nation-specific teachings, worldviews, and philosophies. @CoyoteDreams’ teaching draws an intimate cultural connection between Secwepemc women, waterways, and languages, thus emphasizing that Secwepemc (Indigenous peoples of interior region of British Columbia in Canada) women literally embody their territories and language (Figure 3).

By teaching the language through land-based cultural knowledges, these tweets become a way of digitally rematriating settler-occupied lands, asserting and re-claiming them as Indigenous lands imbued with Indigenous spirituality, knowledges, and relationality. This highlights the potentiality of cyberspace in broader rematriation projects. The extent to which these rematriations materially extend to land is still unknown.

### Digital bridges to homelands and lifeways

For Indigenous language learners who live at a physical distance from their homelands, #NativeTwitter’s language teachings can help to bridge and metaphorically lessen the geographical distances these learners may feel from their communities and homelands. In an interview, Mi’kmaw (refers to a person of the Mi’kmaq people, Indigenous people of the North Eastern Atlantic region of North America) language revivalist Bryson Syliboy, who runs a Mi’kmaq Word of the Day on Twitter, speaks of the digital bridging to homelands and lifeways that Twitter has provided for him: He is currently “living in an isolated community, not on the reserve.” While he emphasizes that learning one’s language within the community itself is vital, he also recognizes that “online is great [. . .] for the fundamentals,” and that engaging with Mi’kmaq through Twitter

brings me closer [. . .] to the community, to the culture. It’s nice to see tweets or videos or pictures of my culture [. . .] It makes you feel less isolated. You can get that inspiration instantly instead of me driving three hours to go to a powwow or go see my family. (Bryson Syliboy, personal communication, 2019)

**Figure 4.**
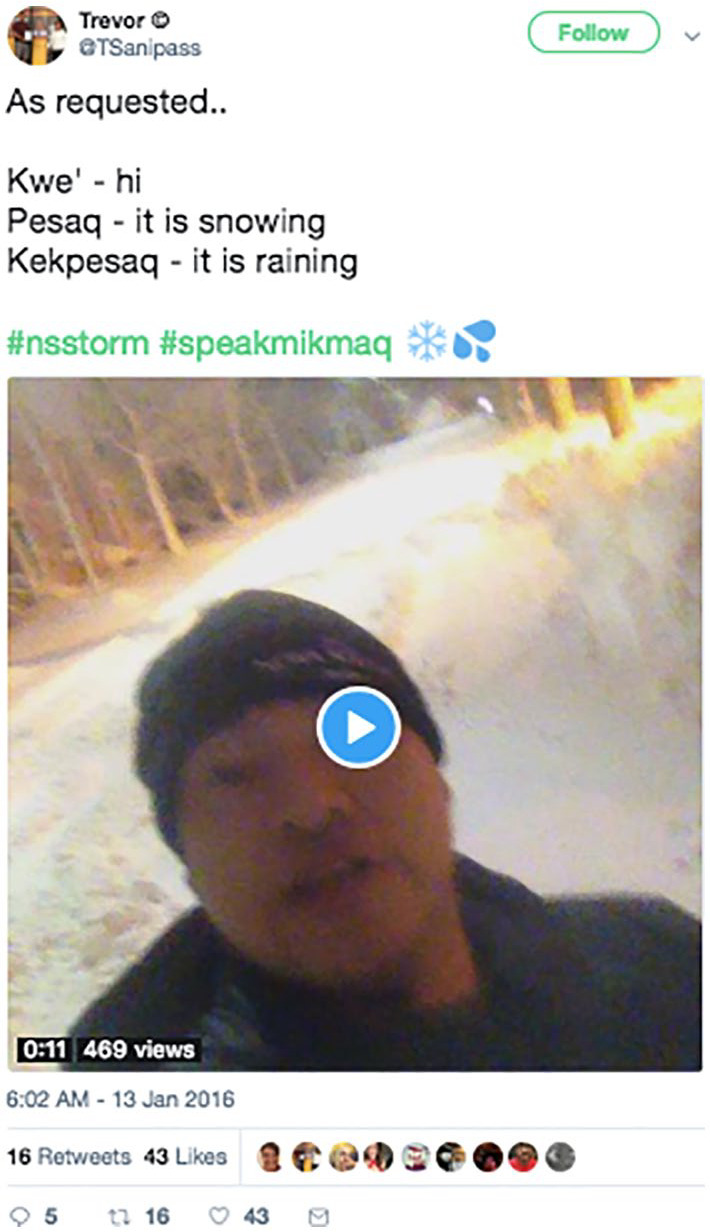
@Tsanipass tweets translations for three Mikmaq words. Mikmaq: Indigenous people of the North Eastern Atlantic region of North America.

Indigenous language revivalists often offer digital bridging by virtually taking their Twitter followers into their homelands through the embedding of photographs and videos of their territory. This immersive land-based experience while learning their language might otherwise be unavailable and inaccessible to Indigenous language learners who reside away from their homelands. As an example, in one of his language tweets, @Tsanipass uses video footage to immerse his followers in a snowy Mi’kmaq (refers to the territory of the Mi’kmaq people, Indigenous people of the North Eastern Atlantic region of North America) landscape while he teaches how to speak about the weather in Mi’kmaq (Figure 4).

**Figure 5.**
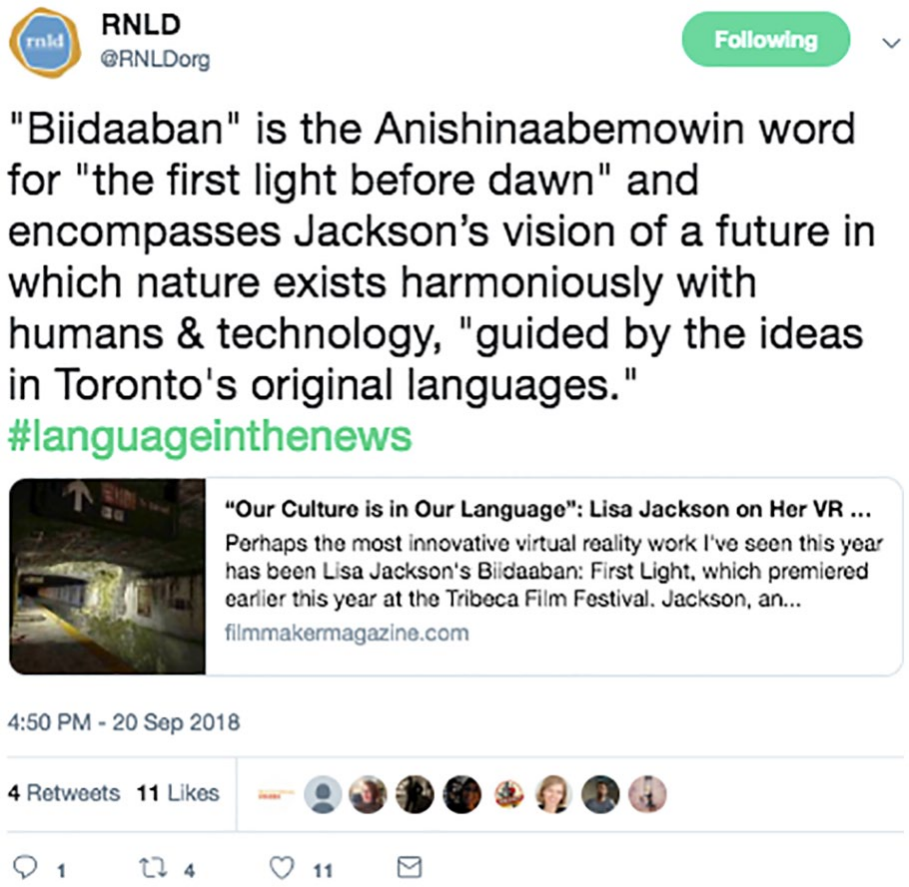
@RNLdorg tweets “the first light before dawn” as a translation for the Anishinaabemowin word “Biidaaban.” Anishinaabemowin: the language of the Annishinaabe people, an Indigenous people in the Great Lakes region of North America.

Digital technologies, especially those which draw on audiovisual capability, make for useful materials which help to bridge Indigenous peoples to places, and particularly ancestral homelands. We see this as especially useful in the context of distance, especially for those who by virtue of either choice or alienation find themselves at great distances from their homelands. While not yet widespread, these immersive engagements by Indigenous peoples to virtually visit their lands are precursors to the possibilities of experiences like that offered by augmented or virtual reality technologies, which simulate geographical places and territories. For example, a project that illustrates the possibilities of simulation technology is Anishinaabe artist Lisa Jackson’s (2018) virtual reality experience *Biidaaban: First Light. Biidaaban* uses virtual reality to offer a new constitution of Indigenous languages, lands, and life. This immersive experience is an assertion that so-called Canadian cities are and always will be Indigenous cities filled with Indigenous knowledges, cultures, and life (Jackson, 2018; K. Todd, 2015). Through this simulation, immersive digital technology enables a holistic, land-based, embodied type of learning and praxis that (a) carries the potential for Indigenous peoples to reconstitute homelands and lifeways significantly altered or under threat of extreme change due to colonial and capitalist forces like climate change, and (b) can also enable acts of neo-constitution, by providing Indigenous peoples the opportunity to explore and claim imagined decolonial futures and lifeways within their territories (Figure 5).

Multi-media embedded into tweets cannot currently offer the 3D experience that *Biidaaban* does. But tweets which use video to take language learners onto the land signify the potentials of Twitter videos for enacting a type of immersive land-based simulation experience. *Biidaaban*’s uses of immersive digital technology can help to further develop and imagine some of the possibilities of embedding videos and other types of multi-media into language-based tweets. Thinking about the types of territorial re-constitution and neo-constitution made possible through these projects can shape how Indigenous Twitter users make films for the purposes of language reclamation on and through Twitter. It is vital to make the distinction that for some Indigenous people, these are re-constitutions, that is, resurgence, reparative, and reclamation efforts, while for others, largely due to colonial history, this is their first engagement with Indigenous language and land.

### Networked cultural navigation

Within Indigenous studies, Twitter has been studied largely as a mobilizing context for social movements and community organizing (Callison & Hermida, 2015; Duarte, 2017; Spears-Rico, 2019), and the proliferation of Indigenous-specific hashtag networks and their global prominence—for example, #MMIWG, #IDLENOMORE—is often framed in this manner. However, recent scholarship speaks to the evolving purposes and ways that Indigenous peoples share their lived experiences and organize as peoples throughout digital ecologies (Molyneaux et al., 2014; Rice et al., 2016) In study, it is clear that #NativeTwitter is filled with hashtag communities dedicated to language learning, such as #SpeakMikmaq, #SpeakGwichinToMe, and #SpeakOjibweToMe. We propose that these hashtag networks are a type of linguistic navigation device. This idea of hashtag networks as tools of navigation is another way that we witness Indigenous land and #NativeTwitter as inextricably interconnected. More specifically, we understand Indigenous language-based hashtag networks as maps and pathways of sorts, which provide language revivalists with roads to homelands and homeland-based supports, communities, cultures, and teachings.

Broadly speaking, maps document human relationships with a landscape, and offer directions to specific destinations within that landscape, while pathways are the roads that one takes to get to these destinations. We see these hashtag networks as decolonial mappings and pathways. Colonial mappings present land in hierarchizing ways that portray land as *a White possession* (Moreton-Robison, 2015) to be conquered, owned, and controlled. On the contrary, Indigenous mappings are infused with kinship and decolonial relationality. Tonawanda Seneca (an Indigenous tribe in New York, USA) scholar Mishuana R. Goeman (2013) suggests that Indigenous mappings and their “narratives . . . mediate and refute colonial organizing of land, bodies, and social and political landscapes” (p. 3). And, through her experiences working alongside the Gitksan (Indigenous people of the Skeena Country in Western Canada), Wet’suwet’en (Indigenous peoples of central interior of British Columbia, Canada), Kaska Dene (Indigenous people in northern British Columbia and southeastern Yukon in Canada), and Gwich’in (Indigenous people in northwestern North America, mostly above the Arctic Circle) nations, Leslie Main Johnson (2010) suggests that Indigenous mappings honour the lived experiences of humans and other-than-humans alike, including bodily experiences, changing seasons, and kinship-making (p. 185). Tewa (a linguistic group of the Peublo peoples, an Indigenous Peoples in southwestern USA) scholar Gregory Cajete (1994) describes Indigenous pathways as “a structural metaphor . . . path denotes a structure; *Way* implies a process” (pp. 54–55).

Indigenous language-based hashtag networks are reflective of these conceptualizations of maps and pathways. As Jackson et al. (2020) write,

Hashtags, which are discursive and user-generated, have become the default method to designate collective thoughts, ideas, arguments, and experiences that might otherwise stand alone or be quickly subsumed within the fast-paced pastiche of Twitter [. . .] creating a searchable shortcut that can link people and ideas together. (p. xxviii)

Among millions of Twitter users and a plethora of hashtags, Indigenous language-learning hashtags are wayfinders to Indigenous cyberterritories imbued with Indigenous life, love, languages, cultures, and futures. Within the White-dominated cyber-realm, these hashtags are virtual Indigenous kinscapes—“storied relations on [and with] the land” (Justice, 2018, p. 197)—that have sparked and fostered nourishing and supportive Indigenous kinship communities for those using Twitter as part of their journey of cultural reclamation. As Black Mi’kmaw (a person of the Mi’kmaq, an Indigenous people of North Eastern Atlantic region of North America) Twitter user Carrington Christmas shared in an interview, through Indigenous Twitter networks,

[y]ou draw [. . .] connections, and you’re able to build meaningful relationships. And I do think digital relationships are a part of real life, you know? I think you may not meet someone in person, but it’s very much still a real relationship and connection that you can have with one another. (Personal communication, 2019)

Some Indigenous language revivalists have even expressed that these kinscapes have inspired them to start their own language-based hashtag movements, which have, in turn, fostered further community-building and cultural reconnection.

Networked cultural navigation is a particularly important feature of digital environments under the shadow of empire. The realities of settler colonial violence have produced various forms of cultural alienation and marginalization, and we see communally produced networks as sites of relational re-constitution which support a resurgence of culture and a revitalization of cultural identity.

## Settler colonialism in cyberspace

There has been increased focus on the colonialism embedded within the manufacturing, construction, labour processes, infrastructures, and systems of digital technologies themselves (Benjamin, 2019; Browne, 2015; Harrell, 2009; Nakamura, 2011, 2014; Noble, 2018; Presner et al., 2014), as well as expanding recognition of how foreign governments and far-right extremist groups have weaponized social media and hashtag networks (Jackson et al., 2020). Research also reveals that Black, Indigenous, and people of colour (BIPOC)—and particularly BIPOC women and BIPOC LGBTQ2IA+ folks—experience high rates of racism and other forms of aggression on Twitter (Daniels, 2017; Dreyfuss, 2018; Jackson et al., 2020; Kim et al., 2018). D. Fox Harrell (2009) writes that “social networking sites, and virtual environments often reproduce forms of social stigma encountered in everyday real life” (p. 49).

**Figure 6.**
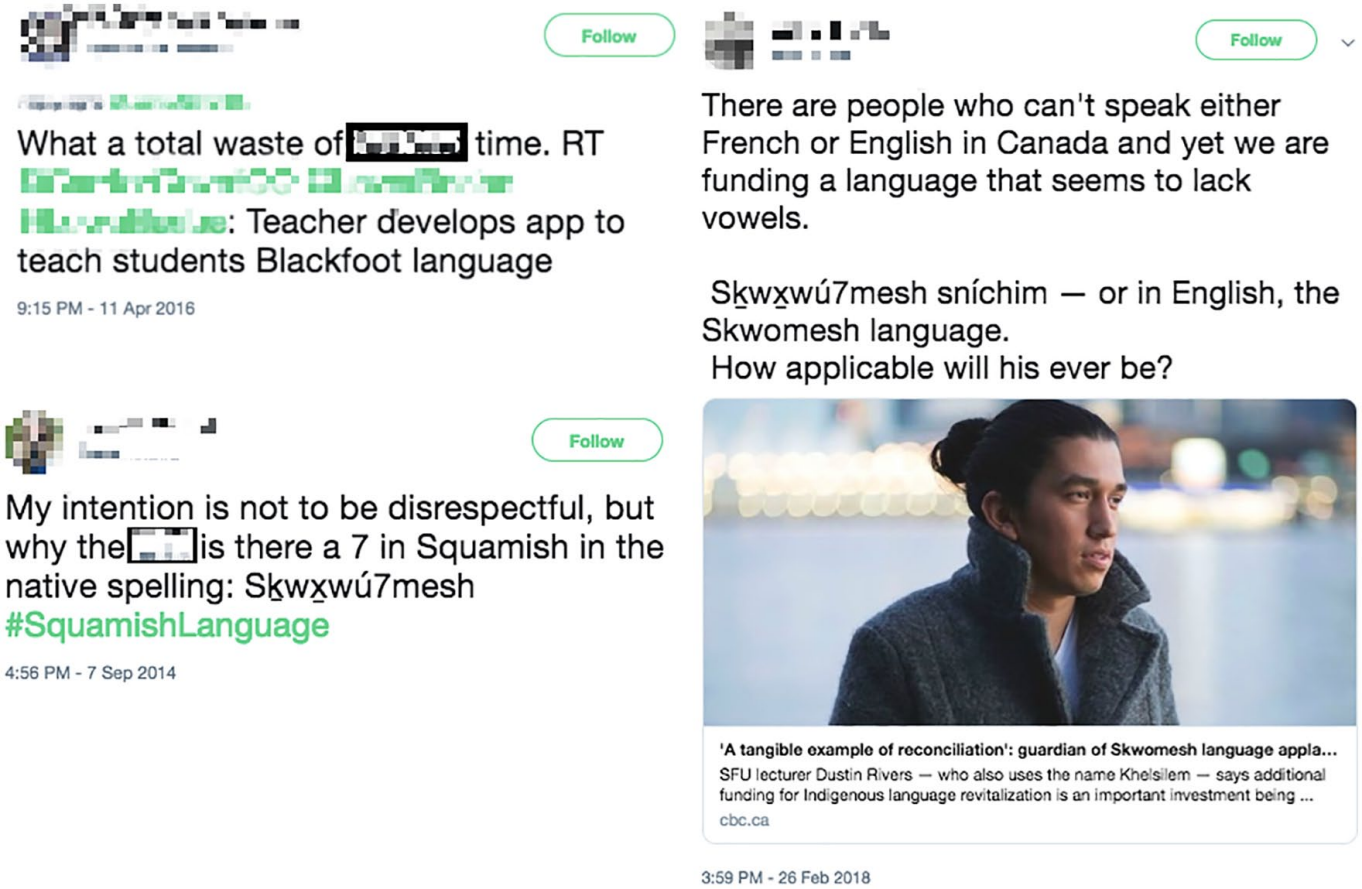
Three tweets showing how land-based colonialism impacts the virtual Twitter space for revitalization of First Nations Indigenous languages. Squamish, Skyxwú7mesh, Skwomesh, Skyxwú7mesh sníchim = Squamish language.

We want to focus on how settlers bring land-based colonialism into the virtual environment of Twitter. As settlers invade and seek to dispossess Indigenous peoples of their lands, settlers often attempt to take over #NativeTwitter and the language, land-based, and cultural teachings occurring in that space. Settlers enact racist discourse and hate speech, steal Indigenous knowledges shared on #NativeTwitter, and commit acts of cultural appropriation (Figure 6).

This coloniality reveals that many non-Indigenous Twitter users fail to be good relations and refuse to recognize or uphold Indigenous protocols when approaching Indigenous knowledge in the Twittersphere. Thus, settler behaviours within Twitter’s ecosystem threaten the Indigenous relationality that weaves through the language-learning occurring within #NativeTwitter.

## Indigenous digital sovereignty and cyber justice

Settler colonialism in cyberspace reveals the connections between issues of land, cultural, and digital Indigenous sovereignty. But, as Indigenous peoples continue to assert their land-based and cultural sovereignty in the face of settler invasions on their homelands, Indigenous Twitter users assert their land-based, cultural, and digital sovereignty within and through #NativeTwitter. The aforementioned #StandWithStandingRock, #IdleNoMore, and #WetsuwetenStrong are testaments of how Indigenous peoples have been asserting their sovereignty and organizing for justice through the platform of Twitter, with Duarte (2017) saying of #IdleNoMore in particular that she “had never seen a transnational Indigenous political movement emerge so quickly through social media networks” (p. 5). The rapid emergence and global reach of these hashtag movements illustrates “that an aspect of Indigeneity, as a paradigm of social and political protest, ha[s] become digitized” (p. 5).

**Figure 7.**
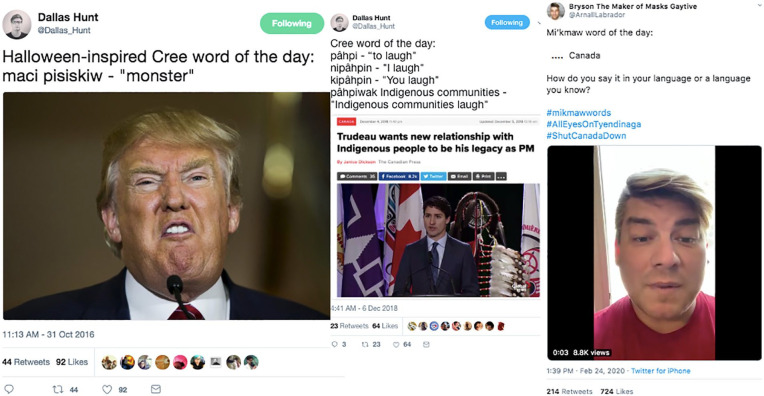
Three tweets showing political themes. Cree = Indigenous people of plains region of North America; Mi’kmaw = a person of the Mi’kmaq people, Indigenous people of the North Eastern Atlantic region of North America.

These assertions of digital sovereignty and online mobilizations for justice are integrally intertwined within the language-learning nests of #NativeTwitter. Indigenous language revivalists on Twitter often use their language posts as a means of drawing attention to the current political sphere. For example, Cree language revivalist Dallas Hunt has spoken out about the White supremacy and colonial violence of both the US President Donald Trump and Canada’s Prime Minister Justin Trudeau through his Twitter-based Cree Word of the Day, while Syliboy has used his Mi’kmaw Word of the Day to speak to Canada’s ongoing colonization of Indigenous lands and life (Figure 7).

Given how settler colonial regimes have attempted to eradicate Indigenous languages from existence through violent policies like the residential school system and the child welfare system, the very act of creating and witnessing tweets written in Indigenous languages is a powerful assertion of ongoing Indigenous sovereignty and survivance. In an interview, Hunt shared the belief that

it’s impossible to do language reclamation work, especially if you’re Indigenous, without it being political in a sense, because you’re speaking back to centuries of colonial violence, and you’re speaking back to it in your language, which has been subject to the various machinations and mechanisms of colonization. (Personal communication, 2019)

Hunt further says that the act of writing tweets in Indigenous languages is “staking a claim to space” (personal communication, 2019)—that is, tweets written in Indigenous languages are overt assertions that Twitter is an Indigenous territory, a space and place of ongoing Indigenous life and cultural revitalization.

The ongoing issue of settler colonialism in Indigenous cyberterritories highlights the need to contend with and address questions of cyber-justice within the Twittersphere, such as, What ethics are implicated through and what protocols are necessary when learning and tweeting one’s ancestral language on Twitter? What happens to local land-based obligations when language revitalization movements are located within transnational digital ecologies like Twitter? How can Twitter be re-programmed to provide a space for Indigenous communities to safely connect and revitalize their languages free from colonial processes? Thinking about the protocols of digital infrastructures, Cree-European artist Archer Pechawis (2014) asks,

What happens when we approach the visioning, creation, and application of modern technology from an entirely Indigenist world view? I am not speaking of grafting Aboriginal protocols onto existing methodologies. I am looking to a future in which Indigenism is the protocol. (p. 38)

Adding to these important questions, we suggest that platforms like Twitter need an honour song. Comanche (Indigenous peoples of southern USA) scholar Dustin Tahmahkera (2021) refers to Indigenous relations with media as “honor scenes”: “Like honor songs for Native Peoples that recognize, respect, and relate Indigenous history, events, and accomplishments, honor scenes engage Natives’ relations with [. . .] media to tell Indigenous-centric stories” (p. 187). Technology needs protocols saturated within Indigenous ethics of relationality, so that, when Indigenous peoples tell their stories online, those stories and the communities related to these stories are kept safe, well-nourished, and respected. We need protocols not merely for the exchange of information, but for ensuring the thriving of people, place, and land.

## Discussion

Our study contributes to a growing body of Indigenous new media research (Duarte, 2017; Gaertner, 2016; Loft & Swanson, 2014; Wemigwans, 2018) that reveals the land-based nature of the Internet. It makes clear that, through digital technology, Indigenous peoples are connecting to their lands in dynamic, culturally grounded, and holistic ways. And it illustrates that Twitter-based Indigenous language-learning networks can be conceived of as navigation and mapping devices that offer digital bridges and pathways to connect language learners with their homelands and communities. This constitutes a novel way of framing Twitter as a technological platform, which, in its repurposing, is strategically useful to the advancement of Indigenous language learning. Indigenous studies scholars (Corntassel & Hardbarger, 2019; McIvor, 2009) have emphasized the integrality of learning one’s language within one’s homeland, and the importance of anchoring language to land-based pedagogies. While it is our perspective that cyberspace can never replace the intimate experience of being on and with land, our analysis highlights that Indigenous language revivalists are offering and practicing land-based cyber-pedagogies which complicate the bifurcation of digital and analogue materiality. That is, our research emphasizes that online learning is not inevitably disconnected from analogue space and place. The ability to connect with one’s homeland through digital means has particularly transformative potential for Indigenous language learners who do not have physical access to their homelands and, thus, must learn from afar. At the same time, there are both technological limitations and political dynamics of technological access that render these potentials as inequitably experienced. This, of course, points to the broader milieu of coloniality within cyberspace.

Our article makes clear that the same colonialism occurring on Indigenous lands plays out in similar and evolving ways, and indeed, as Indigenous new media scholars have suggested, is intrinsically connected to the digital, both in terms of infrastructure and design (Duarte, 2017; L. Todd, 1996). As Sarah J. Jackson et al.’s (2020) research on Twitter suggests, colonial logics extend from the land into the digital environment of Twitter. For one, colonial logics are perceivable in Twitter’s architecture. Our study has documented examples of non-Indigenous users engaging in theft, cultural appropriation, and misuse of Indigenous knowledges within the language-learning networks of #NativeTwitter. Given these acts of coloniality that are playing out in Twitter, our study makes clear that, at the level of design, the public nature of Twitter has real limitations in terms of the protection and stewardship of Indigenous knowledge. Our work joins the scholarship of Anishinaabe (Indigenous people of Great Lakes region of North America) Jennifer Wemigwans (2018) and Gaertner (2018), who have emphasized the risks that open access can pose to the protection of Indigenous knowledges online.

While cyberspace itself is politically occupied, its occupiers are active participants in various forms of exclusion and marginalization. Our study makes clear that colonialism is not only occurring at the structural level, but also plays out intimately in the behaviours of Twitter users. As our research shows, individuals are not only actively engaging in Indigenous knowledge theft, they are also participating in targeted racist and sexist colonial and hate speech within #NativeTwitter’s language-learning networks. These negative engagements and encounters within #NativeTwitter pose a serious risk to the ability of Indigenous users to participate in meaningful and nourishing community-building, cultural revitalization, and language learning within Twitter. While Twitter users from systemically oppressed communities have pointed to the need for increased moderation and suspension of these users (Jackson et al., 2020), we also note the inherent tension of such practices. The scholarship of Jackson, Bailey, and Welles, as well as of Ruha Benjamin (2019) and Simone Browne (2015), point to the ongoing governmental and corporate surveillance of racialized communities through digital technology. If Twitter increases its moderation of users, this increase may also promote and lead to the increased corporate surveillance of systemically oppressed users. On the contrary, our study has also witnessed forms of sousveillance or counter-surveillance occurring within Twitter—that is, surveillance movements led by and conducted at the community-level rather than through the corporation itself: for instance, #SettlerCollector is a hashtag that Indigenous tweeters can use to receive support from non-Indigenous allies when they face racist discourse on Twitter.

Our study makes clear that the Internet, like Indigenous homelands, is a living space not only where ethical relations develop and exist but also where colonial harm to relationships can occur. Protocols are needed and are emerging to respond to moments of coloniality that play out in Twitter’s environment. In line with Wemigwans’s (2018) assertion that Indigenous copyright rules and worldviews should be recognized in cyberspace (p. 44), we feel that Twitter should be re-designed and re-programmed to honour Indigenous epistemologies, protocols, and networks of relationality. We understand the development of these novel approaches to online protocol as digitally constituted forms of anti-colonial resistance and enactments of what we coin cyber-justice in their assertions of digital sovereignty.

To recognize and engage with the Internet as a land-based technology, space, and place offer a stark juxtaposition to and refusal of the “implicit individualism” privileged within colonial epistemologies (Barker, 2010, p. 322). Our study illustrates that Indigenous people are repurposing the Internet, and specifically Twitter, through praxis of anti-colonial kinship and resistance long engaged in various analogue Indigenous spaces. Tuck and Yang (2012) have asserted that “decolonization specifically requires the [concrete, material] repatriation of Indigenous land and life” (p. 21). While the ability of Twitter to enable the material repatriation of Indigenous lands remains to be seen, Indigenous engagements in and implementations of Twitter for language revitalization are supporting and revitalizing Indigenous lifeways: specifically, Indigenous language-learning nests are helping to rematriate culturally specific land-based relationships and knowledges, which emphasize ongoing Indigenous sovereignty; and our analysis suggests that the audiovisual components of Twitter hold the possibility for digital re-constitutions and neo-constitutions of lands and lifeways.

## Conclusion

Throughout this article, we have sought to demonstrate the significance of land-based relations in digital environments, especially Twitter. In framing this work, we have drawn attention to the emerging conceptual and theoretical perspectives offered by Indigenous new media scholars and critical technology theorists who widely critique the landless framing of the Internet. Our study enhances broad theoretical understanding of the interconnectedness of land-based relations in the digital lives of Indigenous peoples, with a focus on the context of language revitalization and survivance. Through our qualitative analysis of language revitalization networks within #NativeTwitter, we make clear that the Internet is indeed land-based, and as such, the politics of settler colonialism and land-based anti-colonial resistance are at work in this space. Our study illustrates that land-based relations occur within cyberspace, and these technological environments provide navigational and mapping traditions for Indigenous peoples to foster these relations. Moreover, Indigenous peoples are creatively navigating settler colonial dynamics within these digital ecologies, all the while enacting anti-colonial resistance. Indigenous peoples’ sovereignty expressed within Twitter points us towards new protocols for cyber-justice, and how Indigenous peoples are repurposing digital technologies guides us towards the type of anti-colonial Internet that is possible. These movements online also signal the needed transformations to current digital environments to bring about an Internet that is conducive to the thriving of Indigenous peoples.

## References

[bibr1-11771801211019097] Barker A. (2010). From adversaries to allies: Forging respectful alliances between indigenous and settler peoples. In Davis L. (Ed.), Alliances: Re/envisioning indigenous-non-indigenous relationships (pp. 331–348).

[bibr2-11771801211019097] Benjamin R. (Ed.). (2019). Captivating technology: Race, carceral technoscience, and liberatory imagination in everyday life. Duke University Press. 10.1215/9781478004493

[bibr3-11771801211019097] Braun V. Clarke V. (2006). Using thematic analysis in psychology. Qualitative Research in Psychology, 3(2), 77–101. https://www.tandfonline.com/doi/abs/10.1191/1478088706qp063oa

[bibr4-11771801211019097] Browne S. (2015). Dark matters: On the surveillance of blackness. Duke University Press. 10.1215/9780822375302

[bibr5-11771801211019097] Cajete G. (1994). Look to the mountain: An ecology of indigenous education. Kivaki Press.

[bibr6-11771801211019097] Callison C. Hermida A. (2015). Dissent and resonance: #Idlenomore as an emergent middle ground. Canadian Journal of Communication, 40(4), 695–716. 10.22230/cjc.2015v40n4a2958

[bibr7-11771801211019097] Corntassel J. Hardbarger T. (2019). Educate to perpetuate: Land-based pedagogies and community resurgence. International Review of Education, 65(1), 87–116. 10.1007/s11159-018-9759-1

[bibr8-11771801211019097] Daniels J. (2017). Twitter and white supremacy: A love story. Dame Magazine. https://academicworks.cuny.edu/hc_pubs/344/

[bibr9-11771801211019097] Dreyfuss E. (2018). Twitter is indeed toxic for women, Amnesty report says. Wired. https://www.wired.com/story/amnesty-report-twitter-abuse-women/

[bibr10-11771801211019097] Duarte M. E. (2017). Network sovereignty: Building the internet across Indian country. University of Washington Press.

[bibr11-11771801211019097] Erb J. Hearne J. Palmer M. with Feeling D. (2018). Origin stories in the genealogy of Cherokee language technology. Boundary, 2. https://www.boundary2.org/2018/07/hearne/

[bibr12-11771801211019097] Gaertner D. (2015). “What’s a story like you doing in a place like this?” Cyberspace and indigenous futurism. Novel Alliances: Allied Perspectives on Literature, Art, and New Media. https://novelalliances.com/2015/03/23/whats-a-story-like-you-doing-in-a-place-like-this-cyberspace-and-indigenous-futurism-in-neal-stephensons-snow-crash/

[bibr13-11771801211019097] Gaertner D. (2016). A landless territory? Augmented reality, land, and indigenous storytelling in cyberspace. In Reder D. Morra L. M. (Eds.), Learn, teach, challenge: Approaching indigenous literatures (pp. 493–498). Wilfrid Laurier University Press.

[bibr14-11771801211019097] Gaertner D. (2018). Towards a methodology of closure. Novel Alliances: Allied Perspectives on Literature, Art, and New Media. https://novelalliances.com/2018/09/25/towards-a-pedagogy-of-closure/

[bibr15-11771801211019097] Goeman M. R. (2013). Mark my words: Native women mapping our nations. University of Minnesota Press.

[bibr16-11771801211019097] Harrell D. F. (2009). Computational and cognitive infrastructures of stigma: Empowering identity in social computing and gaming. In Proceedings of the 7th Association for Computing Machinery (ACM) conference on cognition and creativity (pp. 49–58). ACM Press. 10.1145/1640233.1640244

[bibr17-11771801211019097] Hearne J. (2017). Native to the device: Thoughts on digital indigenous studies. Studies in American Indian Literatures, 29(1), 3–26. 10.5250/studamerindilite.29.1.0003

[bibr18-11771801211019097] Jackson L. (2018). Imagine if Toronto were reclaimed by nature. YouTube. https://www.youtube.com/watch?v=B6EUAEw1-ik

[bibr19-11771801211019097] Jackson S. J. Bailey M. Welles B. F. (2020). #HashtagActivism: Networks of race and gender justice. MIT Press. 10.7551/mitpress/10858.001.0001

[bibr20-11771801211019097] Johnson L. M. (2010). Trail of story, traveller’s path: Reflections on ethnoecology and landscape. Athabasca University Press.

[bibr21-11771801211019097] Justice D. H. (2018). Why indigenous literatures matter. Wilfrid Laurier University Press.

[bibr22-11771801211019097] Kim D. Russworm T. M. Vaughan C. Adair C. Paredes V. Cowan T. L. (2018). Race, gender, and the technological turn: A roundtable on digitizing revolution. Frontiers, 39(1), 149–177. https://www.jstor.org/stable/10.5250/fronjwomestud.39.1.0149

[bibr23-11771801211019097] Lewis J. E. (2014). A better dance and better prayers: Systems, structures, and the future imaginary in aboriginal new media. In Loft S. Swanson K. (Eds.), Coded territories: Tracing indigenous pathways in new media art (pp. 56–77). University of Calgary Press.

[bibr24-11771801211019097] Lewis J. E. Arista N. Pechawis A. Kite S. (2018). Making kin with the machines. Journal of Design and Science. 10.21428/bfafd97b

[bibr25-11771801211019097] Loft S. Swanson K. (Eds.). (2014). Coded territories: Tracing indigenous pathways in new media art. University of Calgary Press. 10.2307/j.ctv6gqr1n

[bibr26-11771801211019097] McIvor O. (2009). Strategies for Indigenous language revitalization and maintenance. In Canadian Language and Literacy Research Network (Ed.), Encyclopedia of Language and Literacy Development (pp. 1–12). Canadian Language and Literary Research Network.

[bibr27-11771801211019097] Molyneaux H. O’Donnell S. Kakekaspan C. Walmark B. Budka P. Gibson K. (2014). Social media in remote First Nation communities. Canadian Journal of Communication, 39(2), 275–288. 10.22230/cjc.2014v39n2a2619

[bibr28-11771801211019097] Monkman L. (2018). Meet some of the influencers of #NativeTwitter. CBC News. https://www.cbc.ca/news/indigenous/indigenous-nativetwitter-influencers-1.4824534

[bibr29-11771801211019097] Moreton-Robison A. (2015). The white possessive: Property, power, and indigenous sovereignty. University of Minnesota Press.

[bibr30-11771801211019097] Nakamura L. (2002). Cybertypes: Race, ethnicity, and identity on the internet. Routledge.

[bibr31-11771801211019097] Nakamura L. (2011). Economies of digital production in East Asia: iPhone girls and the transnational circuits of cool. Media Fields Journal, 2, 1–10. http://mediafieldsjournal.org/economies-of-digital/

[bibr32-11771801211019097] Nakamura L. (2014). Indigenous circuits: Navajo women and the racialization of early electronics manufacture. American Quarterly, 66(4), 919–941. https://muse.jhu.edu/article/563663

[bibr33-11771801211019097] Newcomb S. (1995). Perspectives: Healing, restoration, and rematriation. Indigenous Law Institute.

[bibr34-11771801211019097] Noble S. (2018). Algorithms of oppression: How search engines reinforce racism. NYU Press. 10.1126/science.abm5861 34709921

[bibr35-11771801211019097] Noodin M. Sheldon S. (2017). Waasamodibaajibiigemaazoying: Bright lines of story in song. Studies in American Indian Literatures, 29(1), 88–99. 10.5250/studamerindilite.29.1.0088

[bibr36-11771801211019097] Pechawis A. (2014). Indigenism: Aboriginal world view as global protocol. In Loft S. Swanson K. (Eds.), Coded territories: Tracing indigenous pathways in new media art (pp. 36–47). University of Calgary Press. 10.2307/j.ctv6gqr1n.8

[bibr37-11771801211019097] Presner T. Shepard D. Kawano Y. (2014). Hypercities: Thick mapping in the digital humanities. Harvard University Press.

[bibr38-11771801211019097] Rice E. S. Haynes E. Royce P. Thompson S. C. (2016). Social media and digital technology use among Indigenous young people in Australia: A literature review. International Journal for Equity in Health, 15(81), 1–16. https://equityhealthj.biomedcentral.com/articles/10.1186/s12939-016-0366-0 27225519 10.1186/s12939-016-0366-0 PMC4881203

[bibr39-11771801211019097] Simpson L. B. (2014). Land as pedagogy: Nishnaabeg intelligence and rebellious transformation. Decolonization: Indigeneity, Education & Society, 3(3), 1–25.

[bibr40-11771801211019097] Simpson L. B. (2017). As we have always done: Indigenous freedom through radical resistance. University of Minnesota Press.

[bibr41-11771801211019097] Spears-Rico G. (2019). In the time of war and hashtags: Rehumanizing indigeneity in the digital landscape. In Manjívar J. G. Chacón G. E. (Eds.), Indigenous interfaces: Spaces, technology, and social networks in Mexico and Central America (pp. 180–200). University of Arizona Press.

[bibr42-11771801211019097] Stephenson N. (1992). Snow crash. Bantam Books.

[bibr43-11771801211019097] Styres S. D. (2017). Pathways for remembering and recognizing indigenous thought in education. University of Toronto Press.

[bibr44-11771801211019097] Tahmahkera D. (2021). Honor scenes: Honoring Misty Upham’s critical interventions. Journal of Cinema and Media Studies, 60(2), 187–193. https://muse.jhu.edu/article/781353

[bibr45-11771801211019097] Todd K. (2015). Vancouver is an indigenous city. The Tyee. https://thetyee.ca/Opinion/2015/11/12/Vancouver-Indigenous-City/

[bibr46-11771801211019097] Todd L. (1996). Aboriginal narratives in cyberspace. In Moser M. A. MacLeod D. (Eds.), Immersed in technology: Art and virtual environments. MIT Press. 10.7551/mitpress/3678.003.0012

[bibr47-11771801211019097] Tuck E. Yang K. W. (2012). Decolonization is not a metaphor. Decolonization: Indigeneity, Education & Society, 1(1), 1–40.

[bibr48-11771801211019097] Wemigwans J. (2018). A digital bundle: Protecting and promoting indigenous knowledge online. University of Regina Press.

